# Tandem repeat regions within the *Burkholderia pseudomallei *genome and their application for high resolution genotyping

**DOI:** 10.1186/1471-2180-7-23

**Published:** 2007-03-30

**Authors:** Jana M U'Ren, James M Schupp, Talima Pearson, Heidie Hornstra, Christine L Clark Friedman, Kimothy L Smith, Rebecca R Leadem Daugherty, Shane D Rhoton, Ben Leadem, Shalamar Georgia, Michelle Cardon, Lynn Y Huynh, David DeShazer, Steven P Harvey, Richard Robison, Daniel Gal, Mark J Mayo, David Wagner, Bart J Currie, Paul Keim

**Affiliations:** 1 Northern Arizona University, Center for Microbial Genetics and Genomics, Box 5640, Flagstaff Arizona, 86011 USA; 2 U.S. Army Medical Research Institute for Infectious Diseases, Fort Detrick, Maryland 21702-5011 USA; 3 U.S. Army Edgewood Chemical Biological Center, Aberdeen Proving Ground, Maryland 21010-5454 USA; 4 Brigham Young University, Provo, Utah 84602 USA; 5 Menzies School of Health Research, Charles Darwin University, Darwin, Northern Territory, Australia

## Abstract

**Background:**

The facultative, intracellular bacterium *Burkholderia pseudomallei *is the causative agent of melioidosis, a serious infectious disease of humans and animals. We identified and categorized tandem repeat arrays and their distribution throughout the genome of *B*. *pseudomallei *strain K96243 in order to develop a genetic typing method for *B. pseudomallei*. We then screened 104 of the potentially polymorphic loci across a diverse panel of 31 isolates including *B. pseudomallei*, *B. mallei *and *B. thailandensis *in order to identify loci with varying degrees of polymorphism. A subset of these tandem repeat arrays were subsequently developed into a multiple-locus VNTR analysis to examine 66 *B. pseudomallei *and 21 *B. mallei *isolates from around the world, as well as 95 lineages from a serial transfer experiment encompassing ~18,000 generations.

**Results:**

*B. pseudomallei *contains a preponderance of tandem repeat loci throughout its genome, many of which are duplicated elsewhere in the genome. The majority of these loci are composed of repeat motif lengths of 6 to 9 bp with 4 to 10 repeat units and are predominately located in intergenic regions of the genome. Across geographically diverse *B*. *pseudomallei *and *B*.*mallei *isolates, the 32 VNTR loci displayed between 7 and 28 alleles, with Nei's diversity values ranging from 0.47 and 0.94. Mutation rates for these loci are comparable (>10^-5 ^per locus per generation) to that of the most diverse tandemly repeated regions found in other less diverse bacteria.

**Conclusion:**

The frequency, location and duplicate nature of tandemly repeated regions within the *B. pseudomallei *genome indicate that these tandem repeat regions may play a role in generating and maintaining adaptive genomic variation. Multiple-locus VNTR analysis revealed extensive diversity within the global isolate set containing *B. pseudomallei *and *B. mallei*, and it detected genotypic differences within clonal lineages of both species that were identical using previous typing methods. Given the health threat to humans and livestock and the potential for *B. pseudomallei *to be released intentionally, MLVA could prove to be an important tool for fine-scale epidemiological or forensic tracking of this increasingly important environmental pathogen.

## Background

The environmental saprophyte *Burkholderia pseudomallei *is the causative agent of melioidosis, a disease endemic to tropical regions of Southeast Asia and northern Australia. Symptoms range in severity from fatal sepsis and acute community-acquired pneumonia to benign and localized abscesses. Infection in humans and animals generally occurs through direct contact of open wounds or abrasions with contaminated water and soil, by ingestion of contaminated drinking water, or inhalation of infectious aerosols. Melioidosis is a serious public health threat in Thailand and northern Australia, where it is associated with a case fatality rate of approximately 50 and 20%, respectively [1]. In addition, *B. pseudomallei *has recently attracted attention as a potential biological weapon, and is listed as a Category B biothreat agent by the U.S. Centers for Disease Control and Prevention (CDC) [2].

The close genetic relationship of *B. pseudomallei *to *B. mallei *has previously been demonstrated by DNA hybridization studies [3]. More recently, studies have revealed that *B. mallei *is a clonal lineage of *B. pseudomallei*, and its recent evolutionary divergence is marked by gene deletions and intra-chromosomal rearrangements [4-7]. *B. mallei*, the etiologic agent of glanders, is an obligate parasite of the family Equidae, but can also infect humans through direct contact with infected animals [8] or occupational exposure [9]. Glanders was once a globally distributed disease, but is currently predominant only in the Middle East, Africa, Asia and Central and South America. Due to its highly infectious nature and ability to infect via aerosol, it was used as a biological weapon during World War I and World War II [10,11]. It is also listed as a Category B biothreat agent by the CDC [2].

Due to the severe nature of melioidosis, the molecular epidemiology of *B. pseudomallei *has been investigated using various DNA restriction-based methods, including Pulse Field Gel Electorphoresis (PFGE) [12,13] and ribotyping [14,15]. PFGE has the ability to resolve potentially polymorphic, large DNA restriction fragments, while ribotyping uses restriction fragment length polymorphisms associated with rRNA genes [16]. Although both of these methods have been successful in the epidemiological tracking of pathogens [17], their technical nature can make large datasets more difficult to handle. Also, neither method is easily standardized for transfer throughout the scientific and public health community, and can often lack discriminatory power among closely related isolates within a species or between closely related species [18].

Other procedures that have been used for molecular typing of *B. pseudomallei *involve PCR, such as random amplified polymorphic DNA (RAPD) [19,20] and multilocus sequence typing (MLST) [6]. RAPD detects differences in genomes by amplifying segments of unknown DNA. Drawbacks to this technique include the presence/absence binary nature of the data and the difficulty in reproducing banding patterns between reactions (attributed to PCR artifacts). MLST uses concatenated nucleotide sequences from seven housekeeping genes, that are assumed to be selectively neutral or under purifying selection [21]. This method provides nucleotide data for multiple haplotypes, is easily amenable to phylogenetic analyses and can be standardized across laboratories. The MLST scheme developed for *B. pseudomallei *is also applicable to *B. mallei *and *B. thailandensis*. However, MLST can be time consuming and expensive, and most importantly lacks discriminatory power within closely related *B. pseudomallei *isolates and among the vast majority of *B. mallei *isolates, which are all close genetic relatives [6].

Recently, a reliable PCR-based method using variable-number tandem repeat (VNTR) loci has become a popular tool for the molecular typing of pathogens [18,22-25]. A VNTR locus consists of tandemly repeated sequences of DNA that vary in copy number, creating PCR amplicon size polymorphisms that are easily detected with gel electrophoresis. Due to increased mutation rates when compared to other regions of DNA and their multi-allelic nature, VNTRs allow superior discrimination between closely related isolates. These loci have been successfully implemented for forensic, epidemiological and phylogenetic analyses of bacterial pathogens with low genetic diversity, such as *Bacillus anthracis*, *F*. *tularensis*, and *Y*. *pestis *[23,26-30].

Due to the success of VNTR typing in other pathogens, the primary objective of this study was to develop a high-resolution VNTR typing system for *B. pseudomallei *that is suitable for epidemiological, forensic, phylogenetic and population genetic studies. Thus the first task for this study was to characterize tandem repeat loci, including their distribution and frequency within the *B. pseudomallei *genome. Additionally, in order to develop a comprehensive multiple-locus VNTR typing system that utilizes loci with varying degrees of polymorphism, the second task was to screen loci that were characteristic of the tandem repeat loci throughout the genome and examine levels of polymorphism. Finally, in order to understand the effects that mechanisms such as recombination and mutation have on generating the high levels of diversity observed in this pathogen, it was essential to examine the mutation rates for the non-duplicated VNTR loci chosen for the typing system, as well as a representative sample of the duplicated tandem repeat regions. Furthermore, the estimation of mutation rates will allow for future epidemiological studies that model the transmission of melioidosis in natural populations, similar to published studies on plague [26].

In this manuscript we describe a multiple-locus VNTR analysis (MLVA) genotyping system in which 32 independent, tandemly inserted repeated motifs identified in the *B. pseudomallei *K96243 genome are amplified using fluorescently labeled primers in multiplexed PCRs and separated using capillary electrophoresis. These loci were highly polymorphic across a globally distributed set of 66 *B. pseudomallei *and 21 *B. mallei *isolates, as well as a few very closely related *B. pseudomallei *isolates from an outbreak event and two individual patients.

## Results

### Tandem repeats within the *Burkholderia pseudomallei *genome

**Figure 1:**
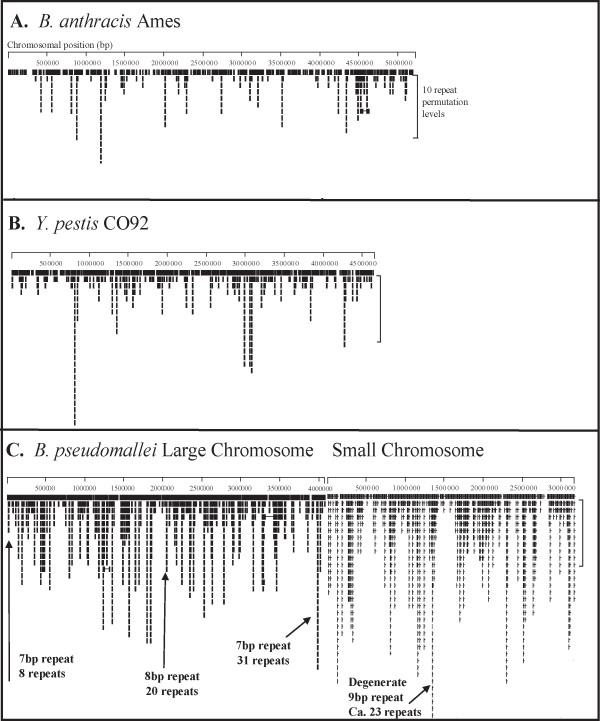
**Linear repeat array distribution of *B. anthracis*, *Y. pestis *and *B. pseudomallei *chromosomes**. Nucleic acid repeat region "icicle" plots were generated with DNAStar GeneQuest software (Madison, WI). The horizontal scale indicates the linear position in base pairs along the respective chromosomes from the start position of the GenBank FASTA file sequence. The scale bar to the right of each icicle plot indicates 10 possible repeat sequence combinations as found by the GeneQuest software. The overall length, or number of possible repeat combinations of each icicle, is a measure of the size of the repeated sequence array found at that position. In general, the longer the icicle, the larger the repeat array. Note that both perfect and degenerate repeat arrays are found and displayed by GeneQuest, as indicated by the arrows and notes in panel C. The number of arrays/Mbp and total arrays are all repeat regions found by the software package Tandem Repeats Finder larger than 30 bp and with an internal similarity greater than or equal to 80%.

**Figure 2:**
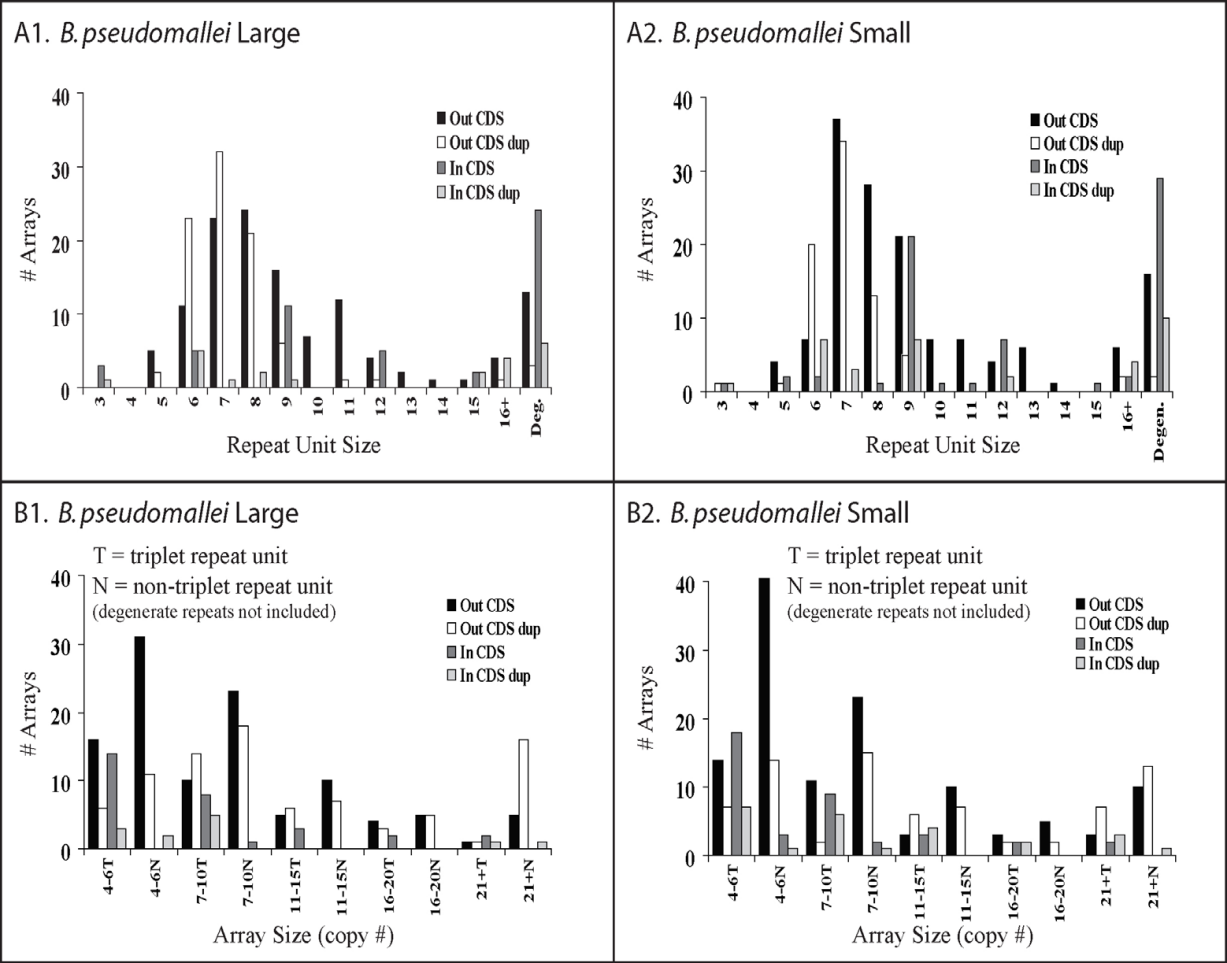
**Repeat region motif size and total array size distribution**. A) Frequencies of arrays consisting of different size repeat motifs in inter-, intragenic and duplicated locations. Degenerate repeats were determined as described in the Materials and Methods Section. B) Frequencies of arrays consisting of different total size classes, again in inter-, intragenic and duplicated locations, based upon triplet and non-triplet repeat motif copy number. Degenerate arrays are not included as consensus repeat motifs were not determined.

We observed that in comparison to other bacterial pathogens with similarly sized genomes, such as *Bacillus anthracis *Ames and *Yersinia pestis *CO92, the *Burkholderia pseudomallei *K96243 genome harbors a relatively large number of tandem repeat arrays (Figure 1). The large (4,074,542 bp) chromosome of *B. pseudomallei *contains 285 (69.9 arrays/Mbp) while the small (3,173,005 bp) chromosome contains 324 (102.1 arrays/Mbp) tandem repeat arrays (Table 1). In contrast, the *Y. pestis *genome contains only 174 arrays and *B. anthracis *contains just 66 arrays, at densities of 37.4 arrays/Mb interval and 12.6 arrays/Mb, respectively. In *B. pseudomallei*, tandem repeat motif sizes on both chromosomes ranged from 3 to 16 bp with copy numbers ranging from 4 to 21 units (Figure 2, A1 and A2). Non-triplet repeat motifs were more common in intragenic regions than inside genes (Figure 2, B1 and B2).

### Distribution and location of tandem repeats

**Figure 3:**
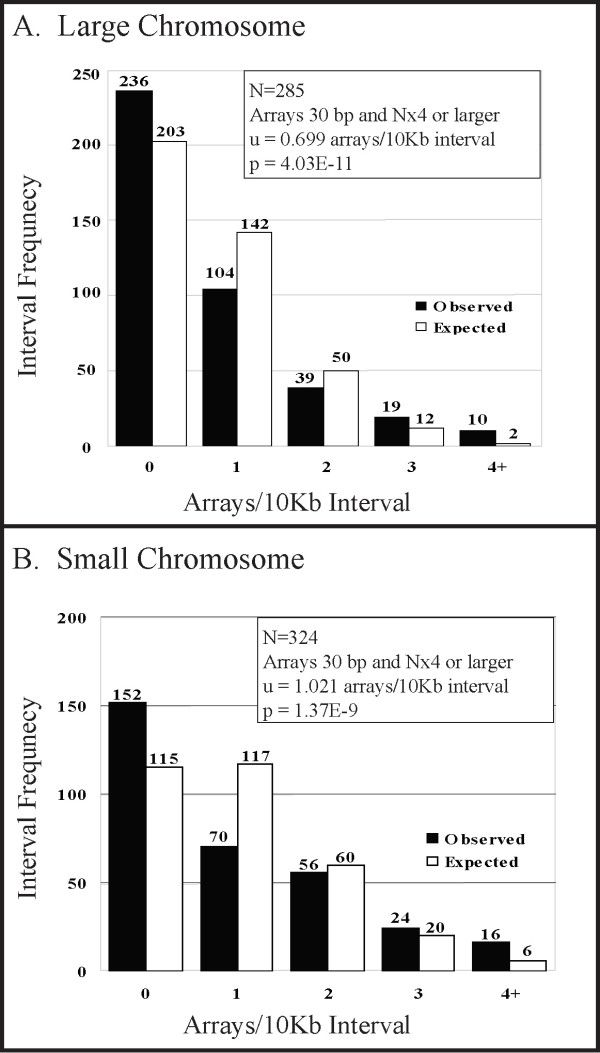
**Repeat array distribution Goodness-of-fit test against a Poisson distribution**. The bar graphs in each of the panels indicate the observed and expected number of 10 Kbp intervals containing zero, one, two, three and four or more repeat arrays for the *B. pseudomallei *large (A) and small (B) chromosomes. For each chromosome, the total number of arrays, average arrays/interval used to generate the Poisson expected frequencies, and calculated p values are shown. Values above each bar indicate the observed or expected frequencies in each category.

A χ^2 ^goodness-of-fit test of the "observed" *B. pseudomallei *tandem repeat distribution to an "expected" Poisson distribution was significant for both the large (p < 0.001) and small chromosomes (p < 0.001) using 10 Kb intervals (Figure 3). The non-random observed distributions for both chromosomes are consistent with a clustered arrangement of arrays throughout both chromosomes. Additionally, the majority of the tandem repeats were found in intergenic regions of the chromosomes: 74.7% (n = 213) tandem repeats on the large chromosome and 68.2% (n = 221) on the small chromosome. However, a portion of these arrays (28.1% on the large chromosome and 35.2% on the small chromosome) were found inside or within 40 base pairs upstream of predicted ORFs (Table 1). Longer arrays (≥ 11 repeat units), including even those with triplet motifs, tended not to be found inside predicted protein coding regions on the large chromosome (Figure 2A1). Conversely, on the small chromosome, longer arrays with triplet repeat motifs were found in both inter- and intragenic locations in almost equal numbers (Figure 2A2). It was also observed that four-fold more degenerate arrays were found on the small chromosome than on the large, and the majority of these degenerate arrays were located inside coding regions (Figure 2A1, and 2A2).

We found that 36.3% of the total number of tandem repeat arrays on both chromosomes of *B. pseudomallei *are duplicated, at least partially (≥ 20 bp and ≥ 80% similarity), in other locations on either chromosome (Table 1). Most of these duplications were found in intergenic regions of the chromosomes and involved the repeat motif only and not the flanking sequences. The majority of duplicated tandem repeats on the large chromosome were, in fact, duplicated on the small chromosome, rather than on the large chromosome. In contrast, arrays duplicated on the small chromosome were found in equal numbers on both chromosomes (Table 1). Additionally, total array lengths were typically longer for duplicated tandem arrays. For example, 104 of the 108 duplicated arrays on the large chromosome, and 112 of the 114 duplicated arrays on the small chromosomes are larger than 200 bp, with the largest almost 6000 bp in size. It was observed that repeat regions that contained more than 20 repeat copies were found to be duplicated in some fashion, and repeat motifs of six and seven bp were more often duplicated than not (Figure 2).

### MLVA development

In order to develop a MLVA system for *B. pseudomallei*, a variety of array sizes were screened, from 2 bp repeat motif by 7 repeat copy unit (i.e. 2 × 7) to degenerate repeat arrays greater than 500 bp but less than 1000 bp, for a total of 104 VNTR loci. We also screened both intra- and intergenically located arrays. Criteria used for including loci in the MLVA system were 1) variation within the screening panel (see Methods), either within the globally distributed or locally distributed outbreak sets, 2) robust (> 80% success) PCR amplification, and 3) highly discrete PCR amplicon sizes (minimal partial repeat differences), based upon locus repeat unit motif. Thirty-two loci met the above three criteria and were chosen for MLVA development (Tables 2 and 3).

### *B. pseudomallei *and *B. mallei *genetic relationships

The 32-locus MLVA system was used to characterize 66 *B. pseudomallei *and 21 *B. mallei *isolates from diverse geographic locations (Table 4). These loci provide high levels of discrimination among different isolates of *B. pseudomallei*, with the number of alleles ranging between 7 to 28, and Nei's diversity values between 0.47 and 0.94 across all *B. pseudomallei *and *B. mallei *isolates (Table 3). Furthermore, the MLVA loci amplified equally well in both *B. pseudomallei *and closely related *B. mallei *strains, and showed variation between and among the two closely related species. MLVA loci did not PCR amplify in the more genetically distant *B. thailandensis *and *B. cepacia*.

**Figure 4:**
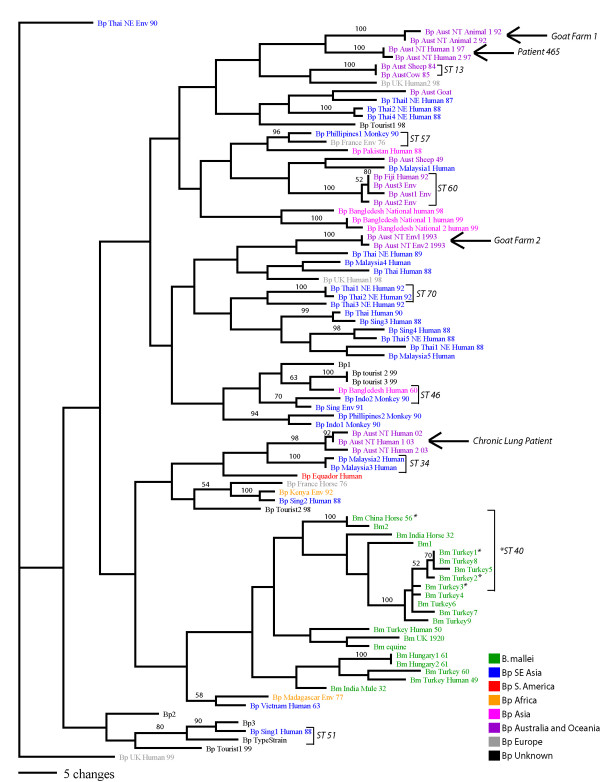
**Arbitrarily rooted phylogram of 66 *B. pseudomallei *and 21 *B. mallei *isolates**. Colors indicate the geographic area from which the isolates were collected. Arrows indicate isolates from patients or from a specific outbreak event. Isolates that had identical MLST genotypes are bracketed and the sequence type is given. * indicates which *B. mallei *isolates were available on the MLST database.

Analysis of allelic variation at 23 loci using a Neighbor Joining distance algorithm revealed 62 genotypes among the 66 *B. pseudomallei *isolates and 19 genotypes among the 21 *B. mallei *isolates. Phylogenetic analysis of these VNTR data provided an extremely high level of strain discrimination even within *B. pseudomallei *isolates from single melioidosis patients (Patient 465 and chronic lung patient) and within isolates from a single *B. pseudomallei *outbreak focus in Australia (Goat Farms 1 and 2) (Figure 4). The average pairwise genetic distance was 0.86 for *B. pseudomallei*, and 0.61 for *B. mallei*.

A phylogram depicting this analysis indicates four highly diverse major clusters among the two *Burkholderia *sp., although there is less than 50% bootstrap support for these branches (Figure 4). These major clusters did not reveal any noticeable geographic or temporal relationships, with isolates from the same country or the same time period occurring in all groups. However, there are many instances in which the relationships between closely related isolates demonstrate clear geographic correlations with solid statistical support (Figure 4). Additionally, the tree indicates that overall, *B. pseudomallei *is much more diverse than *B. mallei*, although this could be due to the less geographically diverse nature of the *B. mallei *isolates. The tree clearly shows that the *B. mallei *isolates form a monophyletic group derived from a *B. pseudomallei *ancestor. The split between *B. mallei *and *B. pseudomallei *is supported by two MLVA loci (3564 k and 2445 k) that contain multiple alleles specific to *B. mallei*.

A comparison of a subset of isolates to other typing methods revealed that MLVA is much more discriminating between closely related isolates. MLST data for 37 of the 66 *B. pseudomallei *and four of the 21 *B. mallei *isolates used in this study were obtained from the online database [31]. A comparison of MLST and MLVA for these 37 *B. pseudomallei *isolates revealed seven instances where MLST sequence types were identical between isolates, while MLVA genotypes were different in all but two of these instances (Figure 4). Of particular note was the single MLST genotype for *B. mallei *and the multiple MLVA genotypes for the same isolates (n = 4). Additionally, a ribotyping study revealed three genotypes for seven of the *B. mallei *isolates (T2, T4, T5, T7, T9, GB5, GB6), while MLVA identified unique genotypes for every isolate [32].

### Mutation rates of tandem repeats

Parallel serial passages experiments (PSPE) from a single *B. pseudomallei *isolate resulted in estimated ~18,000 generations of growth from which lineages were analyzed for variation in all MLVA loci. Mutational events were observed in 12 VNTR loci; the number and type of mutations observed are shown in Table 5. We observed comparable numbers of mutations for loci on each chromosome. There was a noticeable trend towards single repeat mutations (p = 0.0001) as well as bias towards insertion mutations (p = 0.0736) (Table 5). No discernable pattern was observed between loci that had mutations and those without mutations with respect to array size, repeat motif GC %, and/or amplification characteristics. The number of successful lineage PCR amplifications for the mutating MLVA loci ranged from 75–95 (out of 95 possible), averaging 90.25 ± 5.7; while those from the non-mutating loci ranged from 82–95, averaging 92.25 ± 3.1 (data not shown). (The basis of these failures is under investigation, but all mutation rates were corrected appropriately for these missing data.) We observed an average of 1.67 mutations per locus, and mutation rates for individual loci ranged from 5.3 × 10^-5 ^to l.7 × 10^-4^. The combined mutation rate across all 32 loci was 1.113 × 10^-3^, which represents a discrimination power estimator for this MLVA typing system (Table 5). It is similar to the *Y. pestis *MLVA system rate and greater than the *E. coli *rate.

We also examined mutation rates for 17 tandem repeat loci, not included in the final MLVA system, containing arrays found to be duplicated in up to four different locations within and/or between chromosomes (Table 6). In contrast to the MLVA loci, all duplicated loci screened consisted of either six or seven bp repeat motifs, as these were most commonly found with larger duplicated regions in the K96243 strain. Also, while the number of mutations for the duplicated arrays was equal to the MLVA loci, there were more mutations observed for large chromosome loci than small chromosome loci. Additionally, there was a nonsignificant trend towards multiple repeat mutations (p = 0.5127), as well as, a nominally significant trend towards deletion mutations (p = 0.0495) (Table 6). The multiple repeat mutations ranged from 2 to 6 repeat units. Two of the duplicated loci (1558 k and 3851 k), had less than 50% PCR amplification. Highly unpredictable PCR amplification was seen with three loci (3166 k, 1343 k and 2646 k). These PCR failures could be due to the difficult nature of PCR in a high GC organism such as *B. psuedomallei*, or could be indicative of loss of priming sites due to recombination. The PCR amplification success rates for the remaining loci were comparable to the MLVA loci. The duplicated loci averaged 2.6 mutations/locus, and combined mutation rate for 15 duplicated tandem repeat loci was also comparable to the non-duplicated MLVA loci, at 1.23 × 10^-3 ^for ~18,000 generations.

## Discussion

*Burkholderia pseudomallei *is a distinctive microbial pathogen due to its ability to survive and exploit a wide variety of environmental conditions, as well as, the opportunistic infection of animals. It can cause mild, chronic, or rapidly progressing and potentially fatal disease states in a range of animal hosts [33], and it has a demonstrated ability invade the cells of other eukaryotic organisms such as fungi and amoeba [34,35]. It has been known to survive extreme environmental conditions for long periods of time, including nutrient starvation [36], and chlorine concentrations generally recognized as sufficient for potable water treatment [37]. This level of environmental flexibility and pathogenic potential may require the *B. pseudomallei *genome to be highly plastic in order to quickly adapt to different environments. Indeed, while the large chromosome primarily harbors genes essential for growth, the small chromosome contains more diverse genes that are primarily involved in survival and/or exploiting variable or contingent environmental conditions. Consequently, it is not biologically surprising that numerous genetic typing methodologies [6,14,15,22], including the MLVA system reported here, find very high levels of genetic diversity within this organism. The high level of genetic diversity and host flexibility of the organism suggest enhanced mechanisms for generating and maintaining adaptive variation through processes such as selection, recombination and mutation.

The unusually high number of tandem repeats in *B. pseudomallei *(compared to other pathogenic bacteria with similarly sized genomes such as *B*. *anthracis *and *Y*. *pestis*, and other bacteria of similar GC content [5]) is indicative of potentially high genomic diversity which, in turn, may facilitate rapid genomic adaptation to a variable environment. While the majority of large VNTRs in *B. pseudomallei *are located intergenically and thus may have no direct phenotypic effect, it has been observed in other bacteria that such loci, when upstream of genes, can alter important biological functions through mechanisms such as transcriptional regulation and amino acid changes [38-41]. Within coding regions we observed fewer tandem repeat arrays. The majority of these tandem arrays contain repeat units in multiples of three, which indicates the potential for adaptive variation. For example, Nierman et al. [5] observed variation in triplet repeat unit simple sequence repeat (SSR) loci that are located inside four genes coding for surface or putative virulence proteins in *B. mallei *and *B. pseudomallei*. A subsequent serial passage experiment of *B*. *mallei *through several mammalian hosts revealed indels in seven intragenic SSR loci, five of which caused frameshift mutations, while the other two were triplet repeats that only added or removed amino acids from the encoded protein [42]. This variation is consistent with the potential for phase variation during the infection cycle and may be a mechanism to avoid host defenses [5,42]. Thus, given the similarity of *B*. *mallei *and *B*. *pseudomallei*, the unusually high number of tandem repeat loci in *B*. *pseudomallei*, as well as their non-random arrangement, as indicated by a deviation from the expected Poisson distribution (Figure 3), may indicate that coding and non-coding genomic regions use different molecular mechanisms to adapt to different selective pressures.

In addition to the large number of tandem repeats in *B. pseudomallei*, there was a prevalence of duplicated tandem repeats throughout the genome. In *B. pseudomallei*, 37.9% of tandem repeats in the large chromosome and 35.2% of tandem repeats in the small chromosome are found to be duplicated, at least in part, at other intra- and inter-chromosomal locations. Moreover, a serial passage experiment revealed that the duplicated loci show a contrasting trend towards deletions, as well as an increased frequency of multiple repeat changes in comparably sized repeat arrays, while displaying comparable mutation rates to non-duplicated loci; which is in contrast to the lack of bias in *Y. pestis *[43]. This suggests that the repeat regions within *B. pseudomallei *may facilitate large scale genomic rearrangements through recombination rather than slip-strand mispairing [44]. Although this has not been specifically studied in *B. pseudomallei*, it has been suggested that SSRs in *Mycoplasma *genomes may in fact facilitate genomic rearrangements via recombination [45], and that long tracts of tandem repeats may facilitate gene transfer [46]. Conversely, tandem repeats may not directly cause recombination, but rather be associated with regions that are prone to recombination for other reasons. Since recombination frequency is affected by the length of the homology between two loci [47] which in turn is controlled by slip strand repair, the observed tandem repeat patterns could represent an interesting interaction between slip strand expansion and recombination.

During *in vitro *passage, mutation events were observed in multiple *B. pseudomallei *VNTR loci suggesting similar mutation rates at many loci. The MLVA combined mutation rate reported in this study is 1.113 × 10^-3 ^mutations/generation, compared to combined MLVA rates in *E. coli *and *Y. pestis *rates of 6.4 × 10^-4 ^and 1.1 × 10^-3 ^mutations/generation (respectively) [26,43,48]. The combined rate is, hence, comparable to those previously observed in *E. coli *and *Y. pestis *and offers similar subtyping discriminatory power. These rate calculations are dependent upon accurate estimation of the population growth parameters during serial passage and this may be particularly problematic for *B. pseudomallei*, which forms highly mucoid colonies. Experimental serial passage studies in *E. coli *and *Y. pestis *have previously identified a positive correlation between the *in vitro *mutation rate and natural locus diversity. This correlation was not detected in *B. pseudomallei *(analysis not shown) and it is not immediately obvious what differs between these pathogens. Perhaps due the much larger number of VNTR loci in *B. pseudomallei*, the current study was based upon an overwhelming number of equally and highly mutable loci, which are not commonly present in other genomes. In other words, the marker loci in *E. coli *and *Y. pestis *MLVA systems are stratified by their mutability but in the *Burkholderia *MLVA we may examining a number loci that are equally mutable. Thus, there is no correlation with array size. Another interesting difference is in the mutation products, where the majority (19:1) were single repeat changes. This bias was greater than observed in the *E. coli *and *Y. pestis *studies where the single-repeat mutational products were about 80% of the total observed. The lack of more two and three repeat changes needs to be explored in a larger *in vitro *population to see if this trend repeats reality in this particular genome.

Here we present a rapid PCR-based MLVA typing system using 32 independent VNTR loci. Although the initial development of a MLVA system in this organism was complicated by the quantity and duplicated nature of repeated regions found in *B. pseudomallei *and inconsistencies of the allelic size variation in comparison to the repeat unit size, we found 23 markers that were useful for phylogenetic analysis due to high diversity levels, minimal partial repeat differences and amplification success. An additional nine loci, while demonstrating some partial repeat sizes, are very useful for even finer scale resolution of closely related *B. pseudomallei *and *B. mallei *isolates from outbreak situations [49]. While no specific effort was made to design the MLVA primers specific to *B. mallei*, all *B. mallei *isolates tested amplified well at every locus, as expected given the phylogenetic relationship of the two species [6]. Conversely, *B. thailandensis *and *B. cepacia *did not amplify well in any of the loci, indicating that the MLVA loci primers will not support amplification in more distantly related bacterial species, although this has not been explicitly tested. Thus, this MLVA system represents a reliable method of identifying *B. pseudomallei *as well as *B. mallei *strains. Furthermore, this typing method is an easily transferable approach to high-resolution molecular typing analysis using low levels of crudely isolated DNA. The unique size and fluorescent label of each allele, as well as automated sizing software, allows for easy classification of each VNTR allele, and capillary electrophoresis significantly reduces run time.

Due to the relative effects of convergent evolution, reversal mutations, recombination, gene duplications and suggested horizontal gene transfer within *Burkholderia pseudomallei*, phylogenetic hypotheses have been difficult to establish. For example, neither MLST [6] nor MLVA are able to resolve the deeper relationships among distantly related *B. pseudomallei *isolates, as illustrated by the poor bootstrap support for deeper branches (Figure 4) and similar levels of consistency for a subset of the same isolates (~0.63) (data not shown). This lack of resolution results in the absence of a geographic correlation within basal clades, although more derived clades do demonstrate geographic associations between isolates (Figure 4). In comparison, an analysis of Thai and Australian isolates using MLST exhibited no overlap between sequence types for the two countries [50]. However, phylogenetic analysis of these data lacks strong bootstrap values to support this geographic differentiation. Also, the analysis of historical isolates of *B*. *pseudomallei *using MLST reveals an overlapping sequence type between Australia and Thailand environmental isolates, and does not support the genetic distinction of isolates from Australia [51]. Thus, phylogenetic hypotheses using both MLVA and MLST data are difficult to establish with isolates that are geographically and temporally distant.

The present typing system targets VNTR loci over a wide range of diversity levels and consequently provides resolution between *B. pseudomallei *and *B. mallei*, while still providing high levels of discrimination between closely related isolates due to the high variability of tandem repeat loci in these bacterial pathogens. Whereas a number of typing methodologies such as PFGE, ribotyping, RAPDs and MLST have detected differences between isolates, their resolving power among very closely related isolates is less than MLVA [6,14,15,19]. For example, while MLST analysis provided only a single unique genotype for the *B. mallei *cluster, MLVA further resolved the *B. mallei *group into individual genotypes, even among very closely related isolates from Turkey with the same ribotype [32]. Additionally, *B. pseudomallei *isolates with the same sequence type often had different MLVA genotypes (Figure 4). This type of high resolution genotyping can define patterns of mutation within very closely related isolates from an outbreak, which can then be used for generating phylogenetic hypotheses [49].

A recent study by Liu et al. (2006) used six VNTR loci to differentiate *B. pseudomallei *isolates from an outbreak in Singapore [22]. Four of the six loci used were characterized in the present MLVA study. Two of these loci are included in this MLVA (Table 2), but the other two loci were found to be duplicated within the genome, and consequently were not included in MLVA development. This six-locus MLVA offered insight into the epidemiology of *B. pseudomallei *in Singapore, but presented limitations due to the lack of resolution inherent in agarose gel electrophoresis. Given the partial repeat sizes (as small as 3 bp) seen with capillary electrophoresis, it is doubtful that all alleles for these loci were detectable using agarose gels, and thus levels of diversity were underestimated. Additionally, because two of the VNTR loci that were used are duplicated within the genome, they are not recommended for phylogenetic analysis due to the confounding phylogenetic effects of gene duplication and associated possibilities for independent evolutionary trajectories.

## Conclusion

In summary, the findings of this study suggest that the prevalence and location of tandemly repeated regions within the *B. pseudomallei *genome may generate and maintain adaptive variation in this bacterial pathogen. The intragenically located repeat regions, found twice as frequently on the "contingency-oriented" small chromosome [4], may provide for rapid changes in gene function. Duplicated repeat regions may facilitate genomic rearrangements which can lead to altered gene regulation. While the mutation rates of individual repeat regions do not appear to be enhanced over those in other organisms, the sheer number of these regions, some of which are quite large, provides great potential for genetic variation within this species.

Epidemiological characterization is important in any pathogen, but most especially for those that are emerging as global pathogens that may be exploited for biological terrorism, such as *B. pseudomallei*. While no typing system for *B. pseudomallei *can currently be used to reliably establish deep phylogeneic relationships, the *B. pseudomallei*-*B. mallei *multiplex MLVA typing system presented here provides unsurpassed ability to resolve very closely related isolates, even those from the same patient. Efficient and sensitive genetic typing tools, such as the MLVA system presented here, are important for facilitating the increasingly important epidemiological and phylogenetic characterization of emerging pathogens.

## Methods

### DNA preparation

DNA for 66 *B. pseudomallei *and 21 *B. mallei *isolates was obtained from different institutions which used different extraction methods such as Dneasy (Qiagen, Valencia, CA) [52] and phenol/chloroform extraction [32] and quantified using a Pico Green quantification kit (Molecular Probes, Eugene, OR) and a minifluorometer (Turner Biosystems, Sunnyvale, CA). DNA was then normalized to100 pg/μL for VNTR screening. Isolates for the global panel were selected to represent a wide variety of isolates in terms of geographic distribution, host source and date of isolation (Table 4).

### VNTR identification

The complete genome sequence of *Burkholderia pseudomallei *strain K96243 was obtained from the National Center for Biotechnology Information [GenBank: NC_006350, NC_006351] and screened for potentially polymorphic repetitive sequences that were comprised of ≥ dinucleotide repeats, 4 copies and a total array size of 30 bp using GeneQuest (Lasergene, Inc., Madison, WI) and Tandem Repeats Finder [53]. Primers flanking repeat sequences were designed using Primer Express (Lasergene, Inc., Madison, Wis.).

To assess the variability of repeated regions among a globally distributed set of isolates and to develop a comprehensive typing system for this organism, 104 repeated regions (48 from the large chromosome, 56 from the small) were targeted for analysis and subsequent incorporation into a multiple-locus VNTR analysis (MLVA) system. These VNTR loci were selected based upon PCR amplicon size, array size, locus duplication, and proximity to other arrays. Loci resulting in small PCR fragment sizes (<1000 bp) were favored since such loci amplified better than larger regions, and are best suited for analytical platforms. Arrays with fewer than five copies of a motif were not selected for screening. Loci that were duplicated, either within or between chromosomes were also eliminated since multiple alleles would confuse a typing system. Lastly, repeat regions in close proximity (<1000 bp) to other repeat regions were avoided to preserve locus independence. Loci were not excluded based on their intra or intergenic location. The 104 candidate loci were examined for robust amplification and polymorphism across a screening panel which was comprised of 29 *B. pseudomallei *isolates, one *B*. *mallei *isolate (ATCC 10399), and one *B*. *thailandensis *isolate (ATCC 700388). *B*. *pseudomallei *stains in the screening panel included 15 closely related isolates from two different outbreaks in northern Australia [49], and 14 geographically diverse isolates from seven different countries (Table 4). This tiered screening panel allowed us to identify loci with varying degrees of polymorphism.

### VNTR screening using universal tail PCR and genotype analysis

A high throughput five dye Universal Tail amplification and labeling methodology, developed for use in the low GC (x = 35%) bacterium *B. anthracis *[54], was used to screen the chosen repeat region loci for variation among a combination of 29 diverse and closely related *B. pseudomallei *isolates. The optimal Tm for labeling sequences in *B. anthracis *is 55°C, however due to the high G-C (x = 68.12%) content of the *B. pseudomallei *genome, all PCR reactions were performed at a Tm of 72°C.

The UT PCR labeling protocol provides for fluorescent labeling of any PCR amplicon with only four universal fluorescently labeled oligonucleotiodes. The fluorescently labeled universal primer is complimentary to a universal tailed primer sequence on the 5' end of the target specific forward primer (FAM = ACCCAACTGAATAGAGAGC, NED = ATCGACTGTGTTAGGTCAC, PET = CTGTCCTTACCTCAATCTC and VIC = ACGCACTTGACTTGTCTTC). This method significantly reduces the cost of initial screening by not having to order labeled primers for each locus.

PCR amplifications were performed using MJ Research 96-well DNA engines equipped with hot bonnets (Bio-Rad, Waltham, MA). Reaction volumes equaled 10 μL and contained the following: 10× Hot Master Taq buffer with Mg^2+ ^(Brinkmann-Eppendorf, Westbury, New York), 200 μM deoxynucleoside triphosphates (Invitrogen, Carlsbad, CA), 5 μM tailed primer, 50 μM untailed primer, 50 μM fluorescently labeled universal primer (Applied Biosystems, Foster City, CA), 1U Hot Master Taq DNA Polymerase (Brinkmann-Eppendorf, Westbury, New York) and double-distilled H_2_0. After an initial denaturation step at 94°C for 2 min, 30 cycles of touchdown PCR were performed (denaturation at 94°C for 30 sec, annealing for 30 sec with an 0.5°C/cycle decrement at 72°C, and an extension at 72°C for 30 sec) followed by 20 cycles of regular PCR (94°C for 30 sec, 55°C for 30 sec, 72°C for 30 sec), followed by a final extension step for 5 min at 72°C. Negative controls, containing all the components except DNA templates, were included in parallel. PCR samples were stored at -20°C until genotyped.

PCR amplicons were diluted with double-distilled H_2_0 based upon their universal tail sequence (FAM and NED 1:50, PET 1:10 and VIC 1:5) and mixed in equal amounts to provide relatively equal fluorescent signals from each locus during subsequent electrophoresis on an Applied Biosystems 3100 DNA sequencer (Applied Biosystems, Foster City, CA). Size polymorphisms were subsequently analyzed and scored using GeneScan and Genotyper software (Applied Biosystems, Foster City, CA).

### MLVA PCR and genotype analysis

Primers for 32 polymorphic VNTR loci were redesigned with fluorescently labeled forward primers, and optimized for 11 multiplex PCR reactions across *B*. *mallei *ATCC 10399 and the 14 globally diverse *B*. *pseudomallei *isolates used in the initial screening panel (Table 4). These isolates were chosen to increase future amplification success across an array of genetically diverse isolates. MLVA reaction primers (Table 2) were designed to provide uniquely labeled or sized amplicons for every allele at all 32 VNTR loci. PCR amplification of all loci was routinely accomplished using 11 reactions, which were pooled into nine electrophoretic runs.

All reactions contained a final concentration of 1× PCR buffer, 2 mM MgCl_2_, 200 μM of deoxynucleoside triphosphates, 0.08 units Taq DNA Polymerase (Invitrogen, Carlsbad, CA), 1.2 M Betaine (Sigma-Aldrich Co., St. Louis, MO), double-distilled H_2_0, 1 μL of template DNA (~100 pg/μL) and the appropriate primer concentrations for each multiplex PCR (Table 1) for a total volume of 10 μL. Thirteen VNTR loci required the inclusion of a non-fluorescently labeled forward primer in order to decrease the amount of fluorescent amplicon, and thus obtain relatively equal fluorescent signals from each amplicon in the multiplex mix (Table 2). In cases where low DNA quantity affected multiplex PCR results, loci were amplified individually using the same concentrations above and 0.2 μM of both forward and reverse primers.

All PCR reactions were performed in MJ Research 96-well DNA engines equipped with hot bonnets (Bio-Rad, Waltham, MA). PCR reactions underwent an initial denaturation at 94°C for 5 min, 35 cycles of PCR were performed (denaturation at 94°C for 30 sec, annealing for 30 sec at 68°C, and an extension at 72°C for 30 sec) followed by a final extension step for 5 min at 72°C. Negative controls, containing all the components except DNA templates, were included in parallel. PCR samples were stored at -20°C until genotyped.

PCR products for all multiplex mixes were diluted 1:100 with double-distilled H_2_0 and then mixed 1:1 with a 3:1 ratio of formamide to NAU Liz 1007 fluorescently labeled size standard. The PCR products were electrophorectically analyzed with an Applied Biosystems 3730xl DNA sequencer (Foster City, CA). Amplicons were scored using the ABI software program GeneMapper and genotyped according to predetermined allele sizes. An independent party has verified all sizes presented.

### Mutation rate determination

A parallel serial passage experiment used to determine VNTR mutation rates began with a single isolated colony of the Bp9905-1902 strain (T = 0). Bp9905-1902 was a human clinical isolate obtained from the Arizona Department of Health. This colony was dispersed in nutrient broth and then used to start 95 independent clonal lineages by streaking for single colonies on 24 quartered plates. Each lineage was then serially passed 10 times over a 10 day period by streaking a single colony from the previous passage. DNA was extracted from all 95 T = 10 lineages by using an in-house phenol chloroform extraction protocol. PCR for each locus was performed using the universal tail VNTR screening method described above. Mutational events were then visualized using GeneMapper software (Applied Biosystems, Foster City, CA). Using viable plate counts, the number of generations (doublings) per colony was determined to be ~19.93 (log_2 _of the average colony size in cells), which corresponded to a total of 1.81 × 10^4 ^generations in the entire experiment (19.93 generations/colony × 10 passages × 91.03 average analyzed lineages/marker), allowing the detection of mutation rates of 10 ^-4 ^or greater (Table 1). For estimation of cell doubling see discussion and supplemental information in Girard and Wagner et al. [26,24].

### Statistical analyses

Data from 23 loci that displayed greater than 85% amplification success were used to generate an arbitrarily rooted distance-based phylogenetic tree using the Neighbor Joining algorithm in PAUP 4.0 b10 [55]. In order to estimate confidence levels for the analysis, a full heuristic bootstrapping analysis was conducted using a random generator seed for 2000 replicates. Individual marker diversity (*D*) was calculated as equal to 1-∑ (allele frequency)^2 ^and based solely upon allele frequencies in the 87 isolates shown here (Table 1). A χ^2 ^goodness-of-fit test was performed in 10 Kb intervals in order to examine the observed distribution against an expected Poisson distribution for both the large and small chromosomes.

## Authors' contributions

JMU participated in the experimental design, carried out the acquisition of molecular genetic data, data analyses and interpretation and drafted the manuscript. JMS conceived the study and experimental design, carried out the genome survey including the computational and statistical analyses and drafted the manuscript. TP participated in experimental design and edited the manuscript. HH generated the molecular genetic data and edited the manuscript. KLS carried out the serial passage experiment. CLCF, RRLD, SDR, BL, SG, MC and LYH generated the molecular genetic data. DD, SPH, RR, DG and MM carried out the genomic DNA preparation. BJC provided epidemiological and clinical data. DW and PK obtained the funding, conceived the study, helped participate in its design and helped to draft and edit the manuscript. All authors read and approved the final manuscript.

## Figures and Tables

**Table 1:** Summary of *B. pseudomallei *chromosomal repeat region frequency, duplication and location in coding regions

**Chromosome**	**Size (bp)**	**GC%**	**Tandem repeat (TR) regions‡**	**TR/10 kb**	**#TR's in Sanger CDS**	**#TR's within 40 bp 5' of Sanger CDS**	**#TR's within 40 bp 3' of Sanger CDS**	*cis *only duplicated TRs (in CDS)*	*trans *only duplicated TRs (in CDS)*	Both cis and *trans *duplicated TRs (in CDS)*	total dup arrays (in CDS)*
Large	4,074,542	67.7	285	0.699	72**	8	43	22 (4)	56 (12)	30 (2)	108 (18)***
Small	3,173,005	68.5	324	1.021	103**	11	42	25 (8)	48 (14)	41 (13)	114 (35)***
Total	7,247,547	68.1^†^	609	0.860^†^	175**	19	85	47(12)	104(26)	71(15)	222(53)***

‡ Regions with repeats ≥ 2 bp, ≥ 4 repeat units and array sizes ≥ 30 bp

† Average number

*duplications ≥ 20 bp and 80% similarity

**all but 4 and 8 (X1 and X2, resp.) of the non degenerate arrays had RU sizes of 3 bp multiples

***Average duplication size of 50 bp

**Table 2:** VNTR primer sequences and concentrations

**Locus Name**	**Primer Sequence**	**PCR Mix**	**Final [Primer] uM**	**Dye**
**933 k**	F: atggtggcggccgtcggcgaaaacc	1.1	0.20 *	Fam
	R: gctcgaatgggtgtacgaagggccacgctgattc		0.2	
**2065 k**	F: gggggacccggcgcacgacagg	1.1	0.20**	Vic
	R: cggcgcgttgggacgatcggcttgat		0.2	
**2971 k**	F: gcgcaagcgcgactcggccactcg	1.2	0.1	Pet
	R: gtcgccgggcgcggggctacatcttctta		0.1	
**3145 k**	F: ggcaggcaccgccggcatggaagc	1.2	0.2	Ned
	R: gcgtcgcgcgtatcgatccgactgattgtacc		0.2	
**2666 k^b^**	F: gctgcaagtccgccttcacgcgcatcag	2	0.13	Ned
	R: gcggcggccggctcgagttggact		0.13	
**3671 k^a^**	F: gcagcggctttggatcgcccgggttct	2	0.10*	Pet
	R: gggccggggcgcggaagtcgaaagtt		0.1	
**2115 k^a^**	F: ggtgcgtgctggtgtcgctgctgtgctatctgt	2	0.1	Vic
	R: ggggaaggcgccggattgcccgagtt		0.1	
**2341 k**	F: ggcttcgcacccgccccatttcagc	2	0.10**	Fam
	R: gcaccgggcgcggcgcactcg		0.1	
**1500 k**	F: cagagcgcggcgaggacgatcaaaaggag	2	0.10**	Fam
	R: gccgcggctactggcgccaccattg		0.1	
**3091 k**	F: aattcgtcggcagcgggcacggaagatg	3	0.20*	Vic
	R: agcgggcacgcagcttgacggaacc		0.2	
**3152 k^c^**	F: cggcgcggcgttcgtccggctactc	3	0.2	Pet
	R: acgaatgcggggcccgaggttgacgatagg		0.2	
**3652 k**	F: gattcggacggtcggccccgggtatcaa	3	0.25	Ned
	R: gctggacgaaatccggggcgggacaaag		0.25	
**3564 k**	F: ggccatgccgctgccgggttgagc	3	0.20*	Fam
	R: cgcgggaagcgggttttgacgaagggtgtagttt		0.2	
**20 k**	F: gcaccgcgagcgccgagcccgaac	4	0.20 *	Ned
	R: gcgcccggcggccaaccctttgtcg		0.2	
**857 k**	F: cgcgccggatacgccgtccaccag	4	0.2	Fam
	R: acgccggcgccgcaatggctgtc		0.2	
**1690 k**	F: cgtttcccgtttgatgcatttgcgttccctttgaa	4	2	Pet
	R: catcgcggccgtcagaaaagttgagaaacctcgtc		2	
**2445 k**	F: caggccgggccgtcgacgtgttcg	4	0.1	Vic
	R: atcggggagggagggcgacgaggtgaagg		0.1	
**1367 k^a^**	F: ggcgctgccgtggccggacgac	5	0.3	Ned
	R: gccggcgaagcatcgaggcggtatg		0.3	
**1764 k**	F: acccggtcggcacgctacggaactggttgtt	5	2	Pet
	R: cggcggtgaactggcttggcggacctc		2	
**2815 k**	F: cgaggacgcggctcaggtcgatgattttcagg	5	0.1	Fam
	R: cggcgggcgggctttgcatgtcgt		0.1	
**2170 k**	F: cgcatcggcgcaacgtcgtcatctcgt	6.1	0.10*	Fam
	R: cggcgaccgcgcagggcagttga		0.1	
**389 k**	F: gttacaagcgcgggtcggcaagaggctgaaa	6.1	0.10*	Vic
	R: gccggtgttgaacgagtgggtggcgtaagc		0.1	
**1788 k**	F: gcgcggcgagaacggcaagaacgaa	6.2	0.10*	Pet
	R: gagcatcgggtgggcggcgcgtattgat		0.1	
**1217 k^a^**	F: gcgagatgcgggcgtgtgcggtgtg	6.2	0.2**	Ned
	R: gcggcggccgtgagcctgctgagaatc		0.2	
**397 k**	F: cgcacgcgggcaggccgagacg	7	0.20**	Fam
	R: gcggtcgcgcccttccacgcttcatc		0.2	
**2050 k**	F: ccggcggccgcttcgtcgtctcg	7	0.2	Pet
	R: cgcgaagtcgatccgcaactgcctgctcac		0.2	
**2862 k^a^**	F: gattcggcgcggtccgtaccagcttgttgc	7	0.3	Vic
	R: gcgcggggtatgtgacggggcagagc		0.3	
**140 k^a^**	F: gcgcgcaccggccgcttcgactgacga	8	0.3	Fam
	R: gcatacggtcgcgccgggcgggtggtaggaag		0.3	
**2356 k**	F: ccgctgatcggcgtgctgacggtgtt	8	0.2	Ned
	R: gctcggggcgctcggcgttctctg		0.2	
**2518 k^a^**	F: caggcgcagttgtcgattgacgggtgtggac	8	0.2	Vic
	R: acggcgggatgtgcgcggtctgacg		0.2	
**2124 k^a^**	F: ctgcgcgtgctgcccggcgtcac	9	0.2	Vic
	R: cgcgtggcggaatgcgcatgatagg		0.2	
**1934 k^c^**	F: cgacgtgatccgcggctatctcgaagacg	9	0.2	Pet
	R: ccgacgcggcttgccagcttggatcgttag		0.2	

* 50% unlabeled Forward primer

** 75% unlabeled Forward primer

^a ^Not recommended for globally diverse isolates

^b ^Not used in phylogenetic analysis due to < 80% amplification

^c ^Locus reported in Liu et al. 2006 [22]

**Table 3:** MLVA loci characteristics

**Chromosome**	**VNTR Locus Name**	**Array start position in K96243**	**Consensus Repeat sequence**	**In CDS**	**Array Size in K96243 (bpxcopy#)**	**Amplicon Size Range (bp)**	**Number of Alleles**	**Nei's Diversity**
Large	**933 k**	933861	CGGCGAGGGAAA	no	12 × 10	160–365	16	0.89
Small	**2065 k**	2064726	TCGAGTCA	no	8 × 8	238–370	21	0.9
Large	**2971 k**	2971247	CGTGCTT	no	7 × 9	201–314	22	0.92
Large	**3145 k**	3144932	CCTTCCTCG	no	9 × 8	220–345	14	0.86
Large	**2666 k**	2666129	CTTTCGCTA	yes	9 × 7	268–332	8	0.79
Large	**3671 k**	3671327	CTTGGAC	no	7 × 21	205–364	23	0.93
Small	**2115 k**	2115424	CGCCGGTT	no	8 × 15d	290–399	15	0.83
Large	**2341 k**	2340566	TTCGTGCGC	no	9 × 7	122–219	10	0.8
Large	**1500 k**	1500968	GGGAAAGTGCG	no	11 × 6	312–379	7	0.55
Small	**3091 k**	3091444	TCACGGC	no	7 × 12	202–287	11	0.86
Large	**3152 k**	3152382	GACTCG	no	6 × 17	160–371	26	0.94
Large	**3652 k**	3651903	CCGTAGTC	no	8 × 8	320–408	13	0.87
Large	**3564 k**	3563188	GCAGCCTTCTTCGCG	yes	15 × 30d	295–692	10	0.63
Large	**20 k**	20292	CGCCTCA	no	7 × 10	245–435	22	0.92
Small	**857 k**	857207	CGAAYGAGC	no	9 × 11	209–300	12	0.81
Small	**1690 k**	1689945	CGTCGATA	no	8 × 13	252–405	13	0.78
Small	**2445 k**	2444540	GGCACTTC	no	8 × 19	205–391	19	0.89
Small	**1367 k**	1366924	CGCRTCGAA	yes	9 × 24	454–686	26	0.92
Small	**1764 k**	1764166	GCCGCTGAAGTT	no	12 × 20	233–466	12	0.47
Large	**2815 k**	2815153	TGGCGTCTT	yes	9 × 7	223–439	19	0.86
Large	**2170 k**	2171435	ATGCCGTGG	no	9 × 24	229–513	25	0.93
Small	**389 k**	388768	GACGAACC	no	8 × 6	224–313	12	0.87
Small	**1788 k**	1788368	GTCGTGCGATCCTGCT	no	16 × 8	203–367	11	0.86
Large	**1217 k**	1217379	CGGACCTAGG	no	10 × 15	357–480	14	0.85
Small	**397 k**	397146	GCCCGAGA	no	8 × 12	226–401	17	0.88
Small	**2050 k**	2049749	CGATGCGGT/GCACCCAAC	yes/yes	9 × 8/9 × 8	377–549	18	0.92
Small	**2862 k**	2861834	CTCGCCTTTG	no	10 × 8	273–422	15	0.88
Large	**140 k**	139952	GCGCCGAA	no	8 × 15	367–675	28	0.93
Large	**2356 k**	2356018	CTTGGCGA	no	8 × 13	236–425	16	0.9
Small	**2518 k**	2517929	CCGCGAT	no	7 × 31	294–394	17	0.92
Small	**2124 k**	2123866	CCTTCGCG	no	8 × 23	332–490	14	0.88
Small	**1934 k**	1933513	CGAGTCGGCGGTT	no	13 × 16	224–645	21	0.91

**Table 4:** *B. pseudomallei *and *B. mallei *isolates

**Species**	**Strain Name**	**Other Identifier**	**Country of Origin**	**Source**	**Site**	**Date**	**Tree Code**
*B. pseudomallei *(66)	PHLS 5*	2002721617, NCTC 8016	Australia	Sheep		1949	Bp_Aust_Sheep_49
	PHLS 91	2002721622, 84–1097	Australia	Sheep	Lung	1984	Bp_Aust_Sheep_84
	PHLS 92	2002721623, 85–1097	Australia	Cow	Spleen	1985	Bp_AustCow_85
	PHLS 83		Australia	Environment	Soil		Bp_Aust1_Env
	PHLS 84*		Australia	Environment	Soil		Bp_Aust2_Env
	PHLS 85		Australia	Environment	Soil		Bp_Aust3_Env
	PHLS 104		Australia	Goat	Lymph node		Bp_Aust_Goat
	146*		Australia	Animal	Right Udder	1992	Bp_Aust_NT_Animal_1_92
	147		Australia	Animal	Med Lymph Node	1992	Bp_Aust_NT_Animal_2_92
	213		Australia	Environmental	Soil	1993	Bp_Aust_NT_Envl_93
	214		Australia	Environmental	Soil	1993	Bp_Aust_NT_Env2_93
	465a		Australia	Human	Blood	1997	Bp_Aust_NT_Human_1_97
	465e		Australia	Human	Sputum	1997	Bp_Aust_NT_Human_2_97
	1459		Australia	Human	Sputum	2002	Bp_Aust_NT_Human_02
	1627		Australia	Human	Sputum	2003	Bp_Aust_NT_Human_1_03
	1628		Australia	Human	Throat	2003	Bp_Aust_NT_Human_2_03
	PHLS 6		Bangledesh	Human		1960	Bp_Bangledesh_Human_60
	PHLS 208		Ecuador	Human			Bp_Equador_Human
	PHLS 68		Fiji	Human	Blood	1992	Bp_Fiji_Human_92
	PHLS 33	2002721630, 7605	France	Environment	Manure	1976	Bp_France_Env_76
	PHLS 24	2002721620, 7641	France	Horse	Stool	1976	Bp_France_Horse_76
	PHLS 4075		Holland (tourist)	Human	Sputum	1999	Bp_tourist_2_99
	PHLS 4152		Holland (tourist)	Human	Cervix	1999	Bp_tourist_3_99
	PHLS 17		Indonesia	Monkey	Spleen	1990	Bp_Indo1_Monkey_90
	PHLS 18*		Indonesia	Monkey	Pus	1990	Bp_Indo2_Monkey_90
	PHLS 3477		Italy (Tourist SE Asia)	Human	Sputum	1998	Bp_Tourist1_98
	PHLS 31*		Kenya	Environment	Water drain	1992	Bp_Kenya_Env_92
	PHLS 25*		Madagascar	Environment	Soil	1977	Bp_Madagascar_Env_77
	PHLS 71		Malaysia	Human			Bp_Malaysia1_Human
	PHLS 72*		Malaysia	Human			Bp_Malaysia2_Human
	PHLS 73		Malaysia	Human			Bp_Malaysia3_Human
	PHLS 79		Malaysia	Human			Bp_Malaysia4_Human
	PHLS 75*		Malaysia	Human			Bp_Malaysia5_Human
	PHLS 9	2002721637, 521	Pakistan	Human		1988	Bp_Pakistan_Human_88
	PHLS 16		Phillipines	Monkey		1990	Bp_Phillipines1_Monkey_90
	PHLS 14		Phillipines	Monkey	Liver	1990	Bp_Phillipines2_Monkey_90
	PHLS 39*		Singapore	Human	Blood	1988	Bp_Sing1_Human_88
	PHLS 36	2002721635	Singapore	Human		1988	Bp_Sing2_Human_88
	PHLS 38		Singapore	Human		1988	Bp_Sing3_Human_88
	PHLS 40		Singapore	Human		1988	Bp_Sing4_Human_88
	PHLS 19		Singapore	Environment		1991	Bp_Sing_Env_91
	PHLS 3584		Sweden (Tourist SE Asia)	Human	Blood	1998	Bp_Tourist2_98
	PHLS 8*		Thailand	Human		1988	Bp_Thai_Human_88
	PHLS 20		Thailand	Human	Blood	1990	Bp_Thai_Human_90
	PHLS 53	2002721633, 307a	Thailand	Human	Urine	1987	Bp_Thail_NE_Human_87
	PHLS 43		Thailand	Human		1988	Bp_Thai1_NE_Human_88
	PHLS 45		Thailand	Human		1988	Bp_Thai2_NE_Human_88
	PHLS 47		Thailand	Human		1988	Bp_Thai4_NE_Human_88
	PHLS 44*		Thailand	Human		1988	Bp_Thai5_NE_Human_88
	PHLS 392		Thailand	Human		1989	Bp_Thai_NE_Human_89
	PHLS 216	2002721626	Thailand	Environment		1990	Bp_Thai_NE_Env_90
	PHLS 110		Thailand	Human	Urine	1992	Bp_Thai1_NE_Human_92
	PHLS 111		Thailand	Human	Blood	1992	Bp_Thai2_NE_Human_92
	PHLS 112*		Thailand	Human		1992	Bp_Thai3_NE_Human_92
	PHLS 98/SID 2953*		United Kingdom	Human		1998	Bp_UK_Human1_98
	PHLS 98/SID 3292*		United Kingdom	Human		1998	Bp_UK_Human2_98
	99/SID 4349		United Kingdom	Human		1999	Bp_UK_Human_99
	PHLS 2889		United Kingdom (Bangledesh national)	Human	Sputum	1998	Bp_Bangledesh_National_human_98
	PHLS 3811		United Kingdom (Bangledesh national)	Human	Abscess	1999	Bp_Bangledesh_National_1_human_99
	PHLS 3871		United Kingdom (Bangledesh national)	Human	Abscess	1999	Bp_Bangledesh_National_2_human_99
	PHLS 3783*		United Kingdom (Tourist SE Asia)	Human	Sputum	1999	Bp_Tourist1_99
	PHLS 35	2002721638, Ducrete	Vietnam	Human		1963	Bp_Vietnam_Human_63
	PHLS 126						Bp1
	ACTC 11668						Bp2
	ACTC 15682						Bm_Hungary1_61
	ACTC 23343			Type strain			Bp_TypeStrain
*B. mallei *(21)	ACTC 10399*	2002721275, GB11, NCTC 10245	China	Horse	Lung	1956	Bm_China_Horse_56
	ACTC 15310		Hungary			1961	Bp3
	NCTC 10229	GB5	Hungary			1961	Bm_Hungary2_61
	NCTC 3708	GB9	India	Mule	Lung	1932	Bm_India_Mule_32
	NCTC 3709	GB10	India	Horse		1932	Bm_India_Horse_32
	NCTC 10260	GB6	Turkey	Human		1949	Bm_Turkey_Human_49
	NCTC 10248	GB4	Turkey	Human		1950	Bm_Turkey_Human_50
	NCTC 10247	GB7	Turkey			1960	Bm_Turkey_60
	NCTC 120	GB3	United Kingdom			1920	Bm_UK_1920
	85_503			Equine			Bm_equine
	86_567		East India	Mule			Bm1
	ISU						Bm2
	Turkey_1		Turkey				Bm_Turkey1
	Turkey_2		Turkey				Bm_Turkey2
	Turkey_3		Turkey				Bm_Turkey3
	Turkey_4		Turkey				Bm_Turkey4
	Turkey_5		Turkey				Bm_Turkey5
	Turkey_6		Turkey				Bm_Turkey6
	Turkey_7		Turkey				Bm_Turkey7
	Turkey_8		Turkey				Bm_Turkey8
	Turkey_9		Turkey				Bm_Turkey9

* Isolates used in the screening panel.

**Table 5:** *B. pseudomallei*^1 ^MLVA loci mutation rate

**Locus Name**	**Chromosome**	**Inside CDS**	Array size*	**Total Number of Mutations**	**Insertions**	**Deletions**	**Single Repeat Changes**	**Multiple Repeat Changes**	Lineages**	**Mutation Rate**
**1788 k**	Small	no	16 × 9	1	-	1	1	-	94	5.3 × 10^-5^
**2862 k**	Small	no	10 × 11	3	3	-	2	1	90	1.7 × 10^-4^
**1367 k**	Small	yes	9 × 22	1	-	1	1	-	75	6.7 × 10^-5^
**3145 k**	Large	no	9 × 13	1	1	-	1	-	84	6.0 × 10^-5^
**2170 k**	Large	no	9 × 12	4	4	-	4	-	89	2.3 × 10^-4^
**1690 k**	Small	no	8 × 13	2	-	2	2	-	93	1.1 × 10^-4^
**933 k**	Large	no	12 × 14	1	-	1	1	-	91	5.5 × 10^-5^
**2065 k**	Small	no	8 × 14	1	1	-	1	-	95	5.3 × 10^-5^
**2050 k**	Small	yes	9 × 16	1	-	1	1	-	93	5.4 × 10^-5^
**2518 k**	Small	no	7 × 22	2	2	-	2	-	90	1.1 × 10^-4^
**3152 k**	Large	no	6 × 25	2	2	-	2	-	95	1.1 × 10^-4^
**2815 k**	Large	yes	9 × 19	1	1	-	1	-	94	5.3 × 10^-5^
**Total**				**20**	**14**	**6**	**19**	**1**	**† 92.50**	**1.113 × 10^-3^**

^1^An isolate from the Arizona department of health (Bp9905-1902) was used for this study.

**Number of lineages successfully amplified out of 95 total

† Average number of lineages to amplify

**Table 6:** *B*. *pseudomallei*^1 ^duplicated loci mutation rates

**Locus Set **	**Locus Name**	**Array start position in K96243**	**Chromo-some**	**Inside CDS**	Array Size *	**Total Number of Mutations**	**Insertions**	**Deletions**	**Single Repeat Changes**	**Multiple Repeat Changes**	Lineages **	**Mutation Rate**
**1**	**1839 k**	1839378	Large	no	7 × 2	1	-	1	1	-	82	6.12 × 10^-5^
**1**	**3166 k**	3166431	Small	no	-	-	-	-	-	-	-	
**2**	**1853 k**	1853384	Large	no	7 × 27	3	-	3	2	1	94	1.71 × 10^-4^
**2**	**2523 k**	2523234	Small	no	7 × 5	-	-	-	-	-	88	
**2**	**817 k**	817412	Small	no	7 × 17	3	-	3	-	3	89	1.69 × 10^-4^
**3**	**1546 k**	1546409	Large	no	6 × 20	-	-	-	-	-	83	
**3**	**2620 k**	2620013	Large	no	6 × 20	3	1	2	3	-	93	1.64 × 10^-4^
**3**	**3451 k**	3451829	Large	no	6 × 27	1	1	-	1	-	94	5.34 × 10^-5^
**3**	**3103 k**	3103500	Small	no	6 × 27	2	1	1	2	-	94	1.07 × 10^-4^
**4**	**200 k**	199721	Small	no	7 × 12	-	-	-	-	-	94	
**4**	**735 k**	734579	Small	no	6 × 9	-	-	-	-	-	92	
**5**	**1880 k**	1879903	Small	no	7 × 6	-		-	-	-	92	
**5**	**3984 k**	3983644	Large	no	7 × 43	7	3	4	3	4	94	3.82 × 10^-4^
**6**	**1558 k**	1558336	Large	no	6 × 11	**-**	**-**	**-**	**-**	**-**	37	
**6**	**1343 K**	1343285	Small	yes	-	**-**	**-**	**-**	**-**	**-**	-	
**7**	**3851 k**	3851246	Large	no	7 × 17	1	**-**	1	-	1	41	1.25 × 10^-4^
**7**	**2646 K**	2646281	Small	no	-	-	**-**	-	-	-	-	

**Total**						**21**	**6**	**15**	**12**	**9**	**† 83.36**	**1.23 × 10^-3^**

^1^An isolate from the Arizona department of health (Bp9905-1902) was used for this study.

*Estimated array size in the *B. pseudomallei *strain used in the mutation rate study

**Number of lineages successfully amplified out of 95 total

† Average number of lineages to amplify
